# Repro-protective activity of amygdalin and spirulina platensis in niosomes and conventional forms against aluminum chloride–induced testicular challenge in adult rats: role of CYP11A1, StAR, and HSD-3B expressions

**DOI:** 10.1007/s00210-023-02788-9

**Published:** 2023-11-01

**Authors:** Fatma I. Abo El-Ela, Amr Gamal, Hossny A. El-Banna, Marwa A Ibrahim, Ahmed H. El-Banna, Abdel-Razik H. Abdel-Razik, Ahmed Abdel-Wahab, Walid Hamdy Hassan, Asmaa K. Abdelghany

**Affiliations:** 1 Department of Pharmacology, Faculty of Veterinary Medicine, Beni-Suef University, Beni-Suef, 62511 Egypt; 2 Department of Pharmaceutics and Industrial Pharmacy, Faculty of Pharmacy, Beni-Suef University, Beni-Suef, Egypt; 3 Faculty of Veterinary Medicine, Cairo University, Giza, Egypt; 4 Department of Biochemistry and Molecular Biology, Faculty of Veterinary Medicine, Cairo University, Giza, 12211 Egypt; 5 Michael Sayegh Faculty of Pharmacy, Aqaba University of Technology, Aqaba, Jordan; 6 Department of Histology, Faculty of Veterinary Medicine, Beni-Suef University, Beni-Suef, 62511 Egypt; 7 Department of Physiology, Faculty of Veterinary Medicine, Minia University, El-Minia, Egypt; 8 Department of Microbiology Mycology and Immunology, Faculty of Veterinary Medicine, Beni-Suef University, Beni-Suef, 62511 Egypt; 9 Animal and Poultry Management and Wealth Development Department, Faculty of Veterinary Medicine, Beni-Suef University, Beni-Suef, 62511 Egypt

**Keywords:** Amygdalin, Fertility traits, Genes, Niosomes, Oxidative stress, Rat, *Spirulina*

## Abstract

The male reproductive system is negatively influenced by Al exposure. Al represented a considerable hazard to men’s reproduction capabilities. Amygdalin (AMG) and *spirulina platensis* (SP) have been considered to have a strong antioxidant and repro-protective activity; also, targeted drug delivery systems called niosomes improve the distribution of water-soluble medications like amygdalin and spirulina. Current study targeted to determine the effectiveness of AMG and SP against negative reproductive impact resulted by aluminum chloride (AlCl_3_) toxicity. Sixty adult male albino rats were separated into 6 groups, including the control group, which received distilled water; AlCl_3_ group, which received AlCl_3_; AMG+AlCl_3_ group, which received AlCl_3_+AMG; AMGLN+AlCl_3_ group, which received AlCl_3_+amygdalin-loaded niosomes; SP+AlCl_3_ group, which received AlCl_3_+SP; and SPLN+AlCl_3_ group, which received AlCl_3_+spirulina-loaded niosomes. All treatments were orally gavaged daily for 5 weeks, and rats were weighed weekly. At the termination of the experiment, some males (three from each group) were used for fertility traits via mating thirty virgin rat females (in a ratio of 1:2 and 2:3 male:female, respectively) followed by recording of birth weights and litter size (number of pups per each female) at birth to assess males’ reproductive capability. Other males were euthanized for collection of serum, epididymal semen samples, and tissue samples for biochemical, sperm evaluation, gene expression, and histopathological measurements. There are a considerable number of negative impacts of AlCl_3_ on male fertility clarified by declined serum testosterone levels; an increased oxidative stress (MDA, TAC); deteriorated semen quality; down-regulation of CYP11A1, StAR, and HSD-3b gene expressions; and testicular tissue degenerative changes. In addition, litter size (number of pups per each female) and birth weights of pups obtained from mated females were affected. AMG and SP treatments, either in niosomal or conventional form, alleviated the AlCl_3_ negative effects by reducing oxidative stress; increasing testosterone levels; improving semen quality; upregulating of CYP11A1, StAR, and HSD-3b gene expressions; and reducing degenerative changes of testicular tissue. Besides, negative reproductive effect was diminished as observed by changes in the litter size (number of pups per each female) and birth weights of pups obtained from mated females. AMG and SP treatments (either in niosomal or conventional form), ameliorated the AlCl_3_ negative effects as they possess powerful antioxidant activity, as well as they have the ability to improve the reproductive activity of affected males.

**Graphical abstract:**

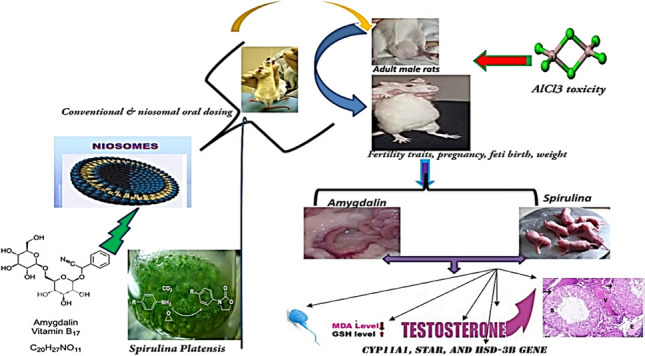

**Supplementary Information:**

The online version contains supplementary material available at 10.1007/s00210-023-02788-9.

## Introduction

Aluminum (Al) is abundant throughout and is detectable in all biological substances and exposure routes. Food, inhalation, and medications are the principal exposure routes for both people and lab animals. Beverages, air pollutants and fumes, medications including ace inhibitors and beauty products, and immunizations containing Al as an adjuvant could be other exposure sources of Al for individuals (Igbokwe et al., 2019; Yokel, 2020). The male reproductive system is negatively influenced by Al exposure. Al represented a considerable hazard to men’s reproduction capabilities. After prolonged exposure, Al accumulates in the testis and, according to the histopathologic investigation, causes obvious lesions in the seminiferous tubules (Yousef & Salama, 2009; Guo et al., 2005). Inflammation in the intertubular compartment of the testis, the emergence of immature spermatocytes in the epididymal lumen, the degeneration of the seminiferous tubules, and a decrease in serum testosterone are also caused by high concentrations of this element (Mouro et al., 2018).

Testosterone is the primary indicator of male reproductive potential; it is necessary for male sexual development, ensuring proper spermatogenesis and the development of secondary sexual characters (Blok et al., 1991). CYP11A1, StAR, and HSD-3b are particular steroidogenic genes required for testosterone production within Leydig cells, and they all play crucial roles in regulating its biosynthesis under the control of luteinizing hormone (LH) (Wang et al., 2017; Zirkin & Papadopoulos, 2018). Few investigations have shown that Al reduces the expression of CYP11A1, StAR, and HSD-3b mRNA (Mohammad et al., 2015; Dong et al., 2016). Furthermore, it has been postulated that the reprotoxic effect of Al could be mediated through induction of oxidative stress in the testicular tissue (Yu et al., 2019). Abnormal generation of reactive oxygen species (ROS) poses a serious threat to sperm cells. Because of their high lipid content, sperm cells are highly susceptible to lipid peroxidation, which causes DNA damage as well as protein denaturation in the cell membrane of sperm and mitochondria (Gharagozloo & Aitken, 2011). In this regard, Al has been found to cause degeneration of seminiferous tubules and spermatogenic epithelium by inducing testicular oxidative damage (Rai et al., 2023). Therefore, in this investigation, we looked at the mRNA expression of such steroidogenic genes and the oxidative state in the testis to identify the molecular mechanism through which Al could have a negative impact on testosterone production and thus sperm quality.

Nanoparticles, such as liposomes and niosomes, offer the potential for more precise and controlled drug delivery (Manosroi et al., 2003; Kazi et al., 2010). Nanoparticle administration improves bioavailability, efficiency, and selectivity of the drug (Manosroi et al., 2003; Kazi et al., 2010; Nowroozi et al., 2018). Niosomes are preferable to liposomes because of the instability of phospholipids when exposed to oxygen and hydrolysis (Manosroi et al., 2003; Kazi et al., 2010). Targeted drug delivery systems called niosomes improve the distribution of water-soluble medications like amygdalin and spirulina by using cholesterol and non-ionic surfactants (Manosroi et al., 2003; Kazi et al., 2010; Nowroozi et al., 2018; Bnyan et al., 2018). Niosomes have been discovered to extend circulation time, lessen toxicity, boost target-site absorption, and improve medication stability (Manosroi et al., 2003; Kazi et al., 2010; Nowroozi et al., 2018).

Because of its unique composition of nutrients and bioactive components (proteins, vitamins, minerals, pigments, and phenolic acids, among others), the unicellular cyanobacterium spirulina platensis (SP) has the potential to be used in a wide range of medical applications (Amor et al., 2017). Phycocyanin, beta-carotene, and other antioxidants and free radical scavengers found naturally in this cyanobacterium have been linked to a reduced risk of cancer and kidney failure (Wang & Zhang, 2016; Hashem et al., 2020). Since SP has been shown to protect multiple organ systems from a wide variety of environmental toxins and heavy metal–induced toxicity, it is not surprising that it is attracting the attention of scientists (Bashandy et al., 2016). It has been reported that SP was effective to protect the reproductive capacity of male mice against bifenthrin-induced reproductive challenges relying to its potent antioxidant activity (Barkallah et al., 2020).

Studies have shown that AMG has positive effects and can be used to cure or prevent a number of illnesses, such as cancer, migraines, chronic inflammation, fever, and pain (Fukuda et al., 2003; Yan et al., 2006). Researchers have previously examined the effects of naturally occurring cyanide-containing chemicals on the male reproductive system, namely, the motility and morphological defects of spermatozoa (Tanyildizi & Bozkurt, 2004). The hyaluronidase activity of spermatozoa was markedly (*P < 0.01*) inhibited *in vitro* when semen samples were treated with amygdalin. In this study, we tried to find out in an vivo study, how it affected the feti birth, number and weight of pregnant rats, and the fertility traits (Jiang et al., 2000).

Reproductive physiology is a complex process that involves dynamic coordination of multiple molecular mechanisms (Ivell & Anand-Ivell, 2021). The dysregulation of the genes involved in the molecular mechanism can functionally disrupt the reproductive physiology (Yatsenko & Rajkovic, 2019) .The current study’s objectives were to evaluate the reprotoxicity of AlCl_3_ in adult male rats using a variety of testicular tissue evaluations, including biochemical biomarkers, gene analysis, histology, and reproductive activity, besides determining the potential protective effects of AMG and SP either in conventional or niosomal forms against the reprotoxicity induced by AlCl_3_. The study presents a comprehensive array of credible evidence to support the idea that AMG and SP may be used as a palliative treatment to prevent infertility in populations exposed to high levels of pesticides containing aluminum chloride.

## Materials and methods

### Chemicals

Agitech pharmaceutical company (Cairo, Egypt) supplied the Span 60, Tween 60, cholesterol, dihexadecyl phosphate, chloroform, and methanol. Aluminum chloride (AlCl_3_) was purchased from an Indian central drug store as aluminum chloride anhydrous (CAS-No: 7446-70-0).

#### Preparation of aluminum chloride

The aluminum chloride (AlCl_3_) powder is freshly prepared by dissolving in distilled water and stored in a dark-colored bottle for oral gavage.

#### Preparation of amygdalin and spirulina platensis powder

Both amygdalin and spirulina were dissolved in distilled water and freshly prepared daily before oral administration to rats.

Amygdalin was obtained as capsule each containing 200 mg of pure amygdalin (99%); B17 with zero filler, preservatives, sugar, sweeteners, chemical. It was manufactured by Amberlotion LLC,USA. 

The green powdered form of Spirulina platensis was obtained from the Amoun Vet. Company (AVC) in Cairo, Egypt, for the synthesis of pharmaceutical medications as a pure green powder.

##### Method of spirulina preparations

After obtaining the green microalgae spirulina platensis, the algae were collected and kept in an alkaline medium pH, and the growth of spirulina usually occurred in the summer, and after its growth, the water is suctioned from the ponds, and the spirulina are collected in a silk cloth that contains small holes in micro size, which filters the algae, and this process is repeated more than once, and it is passed through smaller holes than the aforementioned, then after that it is exposed to a hot air current, then we suction this air in order for the spirulina to dry on this silk cloth, then we collected and grind it, and it is finally squeezed into ultraviolet radiation for sterilization to be ready for use.

After green spirulina preparations, samples are being taken for analysis as shown in Table 1.

**Table 1:** The spirulina analysis with detailed amount  or percentages (%)

Components (green *spirulina platensis*)	Percentages %/amount
Protein	**56.5**
Fatty acid analysis	
Caprylic acid	**1.6**
Capric acid	**34.62**
Myristic acid	**0.41**
Tetradecenoic acid	**0.58**
Pentadecanoic acid	**0.25**
Palmitic acid	**24.64**
Palmitoleic acid	**0.76**
Heptadecanoic acid	**0.21**
Hexadecatrienoic acid	**0.25**
Stearic acid	**1.7**
Oleic acid	**2.29**
Vaccenic acid	**3.14**
Linoleic acid	**12.22**
Gamma-linoleic acid	**11.51**
Eicosanoic acid	**0.24**
	**1.11**
Vitamins	
Vit B2	**0.024 g/kg**
Minerals (%)	
Magnesium	**0.42**
Calcium	**1.08**
Phosphor	**0.93**
Amino acid analysis (%)	
Aspartic	**4.79**
Threonine	**1.24**
Serine	**2.47**
Glutamic	**2.38**
Glycine	**7.34**
Alanine	**2.43**
Valine	**4.63**
Isoleucine	**3.33**
Leucine	**2.79**
Tyrosine	**4.19**
Phenylalanine	**2.23**
Histidine	**0.78**
Lysine	**2.35**
Arginine	**3.03**
Proline	**2.36**
Cysteine	**0.51**
Methionine	**1.24**
Humidity %	**7.7**
Crude fibers %	**3.56%**
Carotenoids	
Vit A carotene %	**98.24 μg/100 g**
Fat %	**1.05**

#### Preparation and optimization of niosome formulation

##### Pre-formulation study

To choose the best niosome formulation for characterization of AMG and SP in vitro and in vivo, pre-formulation investigations were conducted. Effects of different factors with varying molar ratios of dihexadecyl phosphate (DDP) to surfactant (0–0.4), surfactant to cholesterol (0.5–2), and hydrophilic lipophilic balance (HLB) value (4.7–14.9) were analyzed (Kazi et al., 2010; Chauhan & Bhatt, 2019; Chaw & Ah Kim, 2013; Waddad et al., 2013). As dependent variables in this experiment, we assessed the particle size and the entrapment efficiency (%EE). Maximum %EE and minimum particle size were used as optimization criteria. A model medication for pre-formulation experiments was amygdalin.

##### Preparation of niosome formulations

Several different formulations of amygdalin-loaded niosomes (AMGLNs) were made using the thin-film hydration method (Kazi et al., 2010). In a solution of chloroform and methanol (3:1), cholesterol, Tween 60, and dihexadecyl phosphate (DDP) were dissolved. The solution was then transferred to a round-bottom flask and evaporated in a Stuart rotary evaporator (RE300, UK) at 100 rpm, 40 °C, and vacuum. After being dissolved in 10 ml of phosphate buffer, AMG (10 mg) hydrated the film at 60 °C for 2 h (PB). Using a Sonix, AMGLN formulations were ultrasonically treated for 30 min (Illinois, USA). Using a centrifuge (SIGMA, Germany) set at 4 °C and 15,000 rpm for an hour, niosomal pellets were isolated.

##### Determination of entrapment efficiency

Measurements of %EE were used to determine the amount of AMG entrapped in AMGLN formulation (Salem et al., 2018). AMGLN pellets were isolated using a centrifuge (SIGMA, Germany) spinning at 15,000 rpm for 1 h. A UV/Vis spectrophotometer at 255 nm was used to quantify the absorbance of AMG in three replicates (Sohail & Abbas, 2020).

1 $$\% EE=\frac{\left(\textrm{Initial}\ \textrm{AMG}\ \textrm{amount}-\textrm{The}\ \textrm{amount}\ \textrm{of}\ \textrm{AMG}\kern0.5em \textrm{in}\ \textrm{the}\ \textrm{supernatant}\right)}{\textrm{Initial}\ \textrm{AMG}\kern0.5em \textrm{amount}}\times 100$$

##### Particle size determination

The capacity to target the particles depends on their dispersion, homogeneity, and polydispersity index (PDI), which are essential niosome properties (Nowroozi et al., 2018). Dynamic light scattering was used to measure the particle size and PDI in three replicates of each AMGLN formulation after 1-ml samples were diluted with 9 ml of distilled water (Deutches Lokalamt Malvern; DLS) (Gamal et al., 2021).

### In vitro characterization of niosome formulations

#### Transmission electron microscopy (TEM)

The development, surface characteristics, and overall structure of niosome vesicles were studied with transmission electron microscopy (Carl Zeiss, Germany) (Gamal et al., 2021). Using a carbon-coated copper grid, a sample (20 μl) of the AMG-loaded niosome and spirulina-loaded niosome formulations was applied and stained with phosphotungstic dye before being dried.

#### Zeta potential determination images

The electrostatic charge, surface properties, and stability of AMG-loaded niosome and SP-loaded niosome formulations were evaluated by measuring their zeta potential (Bnyan et al., 2018; Chaw & Ah Kim, 2013). Dynamic light scattering (DLS, Malvern, Germany) was used to measure zeta potential in three replicates by mixing a 1-ml sample of each formulation with 9 ml of distilled water (Gamal et al., 2021).

### Animals

Sixty male albino rats weighing 80–100 g were used. The animals were given 14 days to adjust to their surroundings prior to the conduction of the study. A commercial balanced diet was provided, and clean fresh water was available all the day. The microclimatic conditions include temperature, 21 ± 2 °C; relative humidity, 45–60%, and a 12-h light-dark cycle. Twenty adult female rats were used for male fertility assessment in treated rats. The study was performed in guidance with the rules of the local ethical committee, IACUC, Faculty of Veterinary Medicine, Cairo University, with known approval number (Vet CU 03162023640).

### Experimental design

Sixty male albino rats weighing 110–130 g were separated into six groups (gps) (10 rats/each gp) as follows:

1. Control gp: rats received distilled water.
2. Aluminum chloride (AlCl_3_) gp: rats received AlCl_3_ at a dose of 100 mg/kg b.wt. (Yang et al., 2020).
3. Amygdalin+AlCl_3_ gp (AMG+AlCl_3_): rats received 300 mg AMG /kg b.wt. (Song & Xu, 2014).
4. Amygdalin-loaded niosomes+AlCl_3_ (AMGLN+AlCl_3_): AMGLN orally gavaged at a dose of 150 mg/kg. b.wt.
5. Spirulina+AlCl_3_gp (SP+AlCl_3_): rats orally gavaged with SP at a dose of 300 mg/kg b.wt. (Khalil et al., 2018).
6. Spirulina-loaded niosomes+AlCl_3_ gp (SPLN+AlCl_3_): rats administered SPLN orally at a dose of 300 mg/kg b.wt. (Khalil et al., 2018).

In all groups, rats received AlCl3 and treatments daily for 5 weeks by the oral route via gastric gavage. Additionally, the treatments were given before AlCl_3_ administration by 1 h.

### Body weight

Rats were weighed weekly using a digital scale. Weight was recorded, and the weight gain has been estimated by deducting the variation in weight between the start and the end of the experiment.

### Fertility traits

Treated male rats were used for fertility assessment, where three males from each group were used for natural mating of five virgin females in a ratio of 1:2 or 2:3 male:female, respectively. Vaginal smear was examined for detection of sperms, and then mated females were palpated at 12–14 days after mating. Litter size and birth weight of neonates were recorded at birth (Ochiogu et al., 2006; Agematsu et al., 1983).

### Serum and tissue samples

After 35 days, samples of blood were obtained from the rats eyes’ medial canthus in clean tubes without anticoagulant followed by centrifugation at 3000 rpm for 15 min to obtain serum samples that were stored at − 20 °C until use. Then, all rats were humanely killed by decapitation after an overnight fast and ketamine and xylazine combinations for anesthesia. The abdomen was gently opened, and the testes were removed. Then, testes were dissected into three sections, one of which was kept at − 80 °C for gene expression determination. The second portion was placed in 5 mL phosphate-buffered saline (NaCl 8 g/L, KCl 0.2 g/L, Na_2_HPO_4_ 1.44 g/L, and KH2PO4 0.24 g/L) and subjected to homogenization. The homogenates were then centrifuged for 20 min at 11,200 ×g, and the supernatants were obtained to assess the malondialdehyde (MDA) and total antioxidant capacity (TAC). The third portion was fixed in Bouin’s fluid histopathological examination.

### Epididymal semen sample collection and evaluation

Epididymis was placed in a clean worm Petri plate with a few drops of warm physiological saline and dissected. Individual motility, sperm vitality (eosin nigrosine stain), total sperm abnormalities, and sperm concentration were all assessed as described previously (Abdoli et al., 2012).

### Measurement of testicular levels of MDA and TAC

The tissue homogenates were measured using specific rat colorimetric assay kits as directed by the instructions of the manufacturer.

Total antioxidant capacity (TAC): by using the QuantiChromTM Antioxidant Assay Kit (Catalogue number: DTAC-100, Hayward, CA 94545, USA).

Malondialdehyde (MDA): by using kit purchased from Biodiagnostic Company, Egypt (Catalogue Number: MD 25 29).

### Measurement of serum testosterone levels

Serum testosterone levels were measured using a particular Rat Testosterone ELISA Kit (Catalogue number: csb -e05100r; Houston, TX 77054, US.). An enzyme immunoassay technique known as competitive inhibition was used in this test. This kit’s microtiter plate contains the goat-anti-rabbit antibody pre-coated on it. Both the samples and the standard were put into the wells together with a testosterone-specific antibody and tagged testosterone. A competitive inhibitory reaction between HRP-labeled and unlabeled testosterone was started using the antibody. The wells were then filled with a substrate. As the level of testosterone in the sample increases, the color changes in the opposite manner. After stopping the color’s development, the intensity of the color was measured.

### Measurement of the transcript levels Of StAR, CYP11A1, and HSD-3b

With the use of the EasyRNATM Cell/Tissue RNA Mini Kit (BioVision #K1337), total RNA from testicular tissue was recovered. Following the manufacturer’s instructions, SuperScript Reverse Transcriptases (Thermo Fisher Scientific) were used to create first-strand cDNA. On an ABI Prism StepOnePlus Real-Time PCR System (Applied Biosystems), quantitative PCR was carried out using PowerTrack SYBR Green Master Mix in accordance with the manufacturer’s instructions (Noshy et al., 2022). Table 2 contains the primer sets for the evaluated genes, and the expression of the target mRNA was standardized to ACTB.

**Table 2:** The primer sets of the studied genes

	Sense	Antisense	Amplicon	Accession no.
*StAR*	TGGCTGCCAAAGACCATCAT	TGGTGGGCAGTCCTTAACAC	241	NM_031558.3
*CYP11A1*	GCAAAAGGTCTTTGCCTGCG	TGGATTCTGTGTGTGCCGTT	212	NM_017286.3
*HSD-3b*	CTCACATGTCCTACCCAGGC	TATTTTTGAGGGCCGCAAGT	362	NM_001007719.3
*ACTB*	CCGCGAGTACAACCTTCTTG	CAGTTGGTGACAATGCCGTG	297	NM_031144.3

### Histopathological studies

The testicular tissue samples underwent ethyl alcohol dehydration in increasing concentrations, washed in xylene, impregnated in soft paraffin, and then embedded in hard paraffin. Rotatory microtomes were used to cut sections that were 4–6-m thick, which were then put on dry, clear glass slides. Hematoxylin and eosin was used to stain the acquired slides (H&E). The light microscope was attached to an LEICA (DFC290 HD system digital camera, Heerbrugg, Switzerland) employing 10, 20, and 40 objective lenses for histopathology detection (Bancroft & Gamble, 2008).

### Histopathological scoring

Testicular tissue damage was graded according to the following scale: 0 indicates no change, 1 indicates 25% tissue damage, 2 indicates 26–50% tissue damage, 3 indicates 51–75% tissue damage, and 4 indicates 76–100% tissue damage (Gibson-Corley et al., 2013).

### Statistical analysis

The data was analyzed using SPSS version 22 statistical software and one-way analysis of variance (ANOVA) and post hoc test (Dunnett test) were applied for tables. GraphPad Prism10 was used for graphs by one-way analysis of variance (ANOVA), and repeated measures ANOVA, followed by post hoc test (Dunnett test). The data were statistically significant at *P < 0.05* and were shown as mean ± standard deviation of the mean.

## Results

**Fig. 1:**
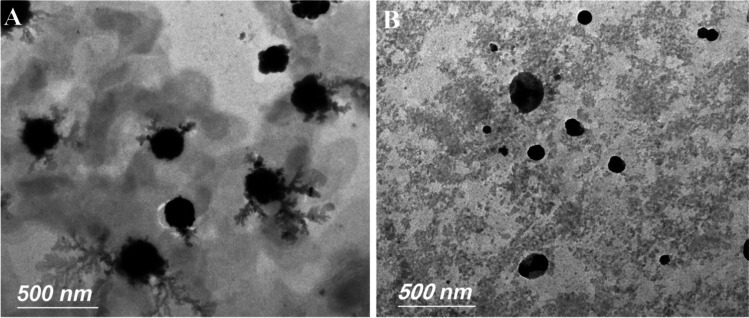
Transmission electron microscopy of AMGLN formulation (**A**) and SPLN formulation (**B**)

Thin-film hydration technique was used to successfully prepare amygdalin-loaded niosome (AMGLN) and spirulina-loaded niosome formulations. The %EE and particle size of different prepared AMGLN formulations were determined to be 9.12 ± 0.72% to 68.32 ± 0.69% and 198.4 ± 6.97 nm to 753.6 ± 8.85 nm, respectively. A pre-formulation investigation revealed that an increase in HLB values was associated with a significant (*p* < 0.05) increase in %EE and particle size. A %EE of 68.32 ± 0.69% and a particle size of 252.63 ± 11.38 nm were observed for a formulation containing Tween 60 (HLB = 14.9):cholesterol: DDP in a molar ratio of 1:2:0.1, while a %EE of 9.12 ± 0.72% and a particle size of 198.4 ± 6.97 nm were observed for a formulation containing Span 60 (HLB = 4.7). According to preliminary research, there is a positive correlation between cholesterol concentration and both %EE and particle size. A %EE of 68.32 ± 0.69% and a particle size of 252.63 ± 11.38 nm were seen in a formulation including Tween 60, cholesterol, and DDP in a molar ratio of 1:2:0.1, while a %EE of 27.13 ± 1.05% and a particle size of 325.15 ± 6.55 nm were observed in a formulation containing Tween 60, cholesterol, and DDP in a molar ratio of 1:1:0.1 at the molar ratio studied, all AMGLN formulations with DDP showed a higher %EE and smaller particle size than those without DDP. The formulation with a Tween 60:cholesterol:DDP molar ratio of 1:2:0.1 was selected after examining particle size and %EE data. The sizes of AMGLN and SPLN formulations were determined and found to be 252.63 ± 11.38 and 282.15 ± 7.75, respectively. The zeta potentials of the AMGLN and SPLN formulations were measured and found to be − 4.82 ± 0.73% and − 6.02 ± 0.88%, respectively. The morphology of the niosome formulations is investigated in Fig. 1. The vesicles showed spherical vesicular structures existing in a dispersed pattern.

**Fig. 2:**
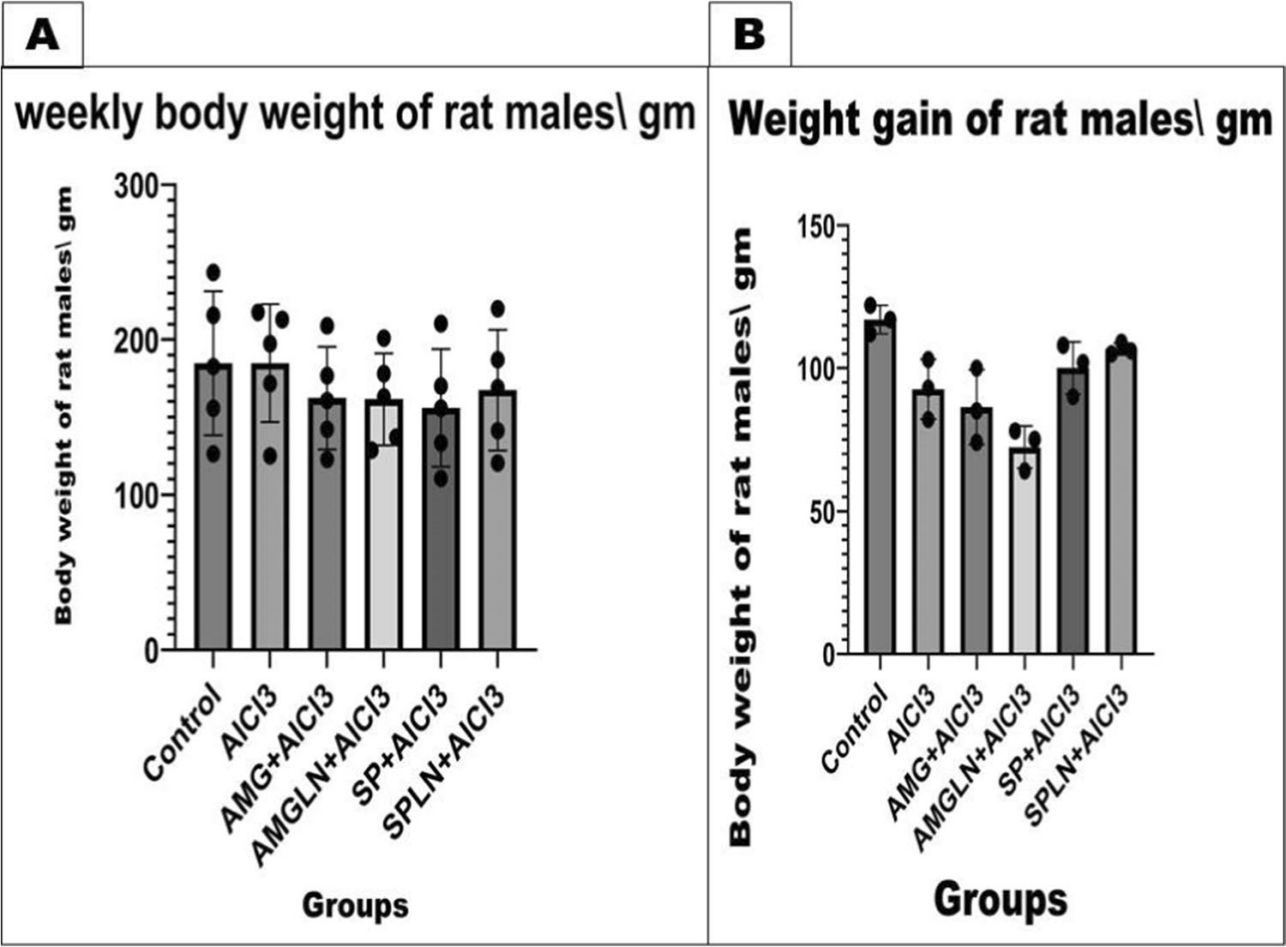
Effect of AlCl_3_ and treatments (AMG, SP) on body weight (**A**) and weight gain (**B**) of male rats. Results presented as mean and standard deviation of mean. Data graphed using GraphPad Prism10 were used for graphs by one-way analysis of variance (ANOVA), and repeated measures ANOVA, followed by post hoc test (Dunnett test)

Figure 2 clarifies a marked reduction in the body weight of rat males (*P < 0.05*) by SP and SPLN treatments when compared to the control males throughout the experimental period, while they improved the rats’ weight with time (weight gain). Moreover, the calculated weight gain showed a considerable reduction in the rats treated by AlCl3 (*P < 0.05*) and AMG+AlCl_3_, and AMGLN+AlCl_3_ treatments (*P < 0.01*). The AlCl3-treated rats had no effect on weekly body weight in comparison with other gps; however, it showed an obvious (*P < 0.05*) decrease in the calculated weight gain in comparison with control rats.

**Fig. 3:**
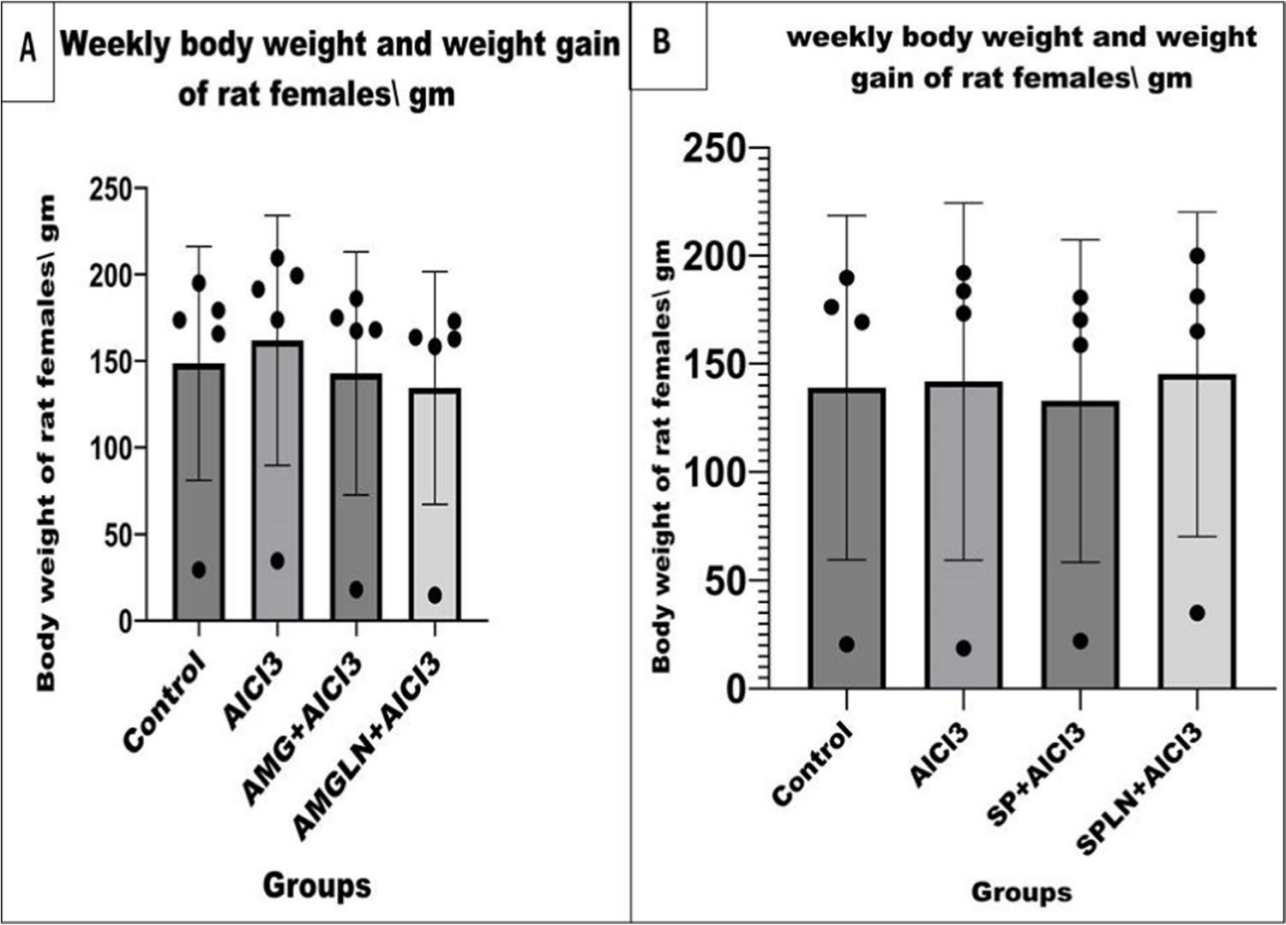
Effect of AlCl_3_ and treatments on body weight and weight gain of mated female rats: **A** control, AlCl_3_-, AMG-, AMGLN-treated mated females, **B** control, AlCl_3_-, SP-, SPLN-treated mated female rats. Results presented as mean and standard deviation of mean. Data graphed using GraphPad Prism10 were used for graphs by repeated measures ANOVA followed by post hoc test (Dunnett test)

**Fig. 4:**
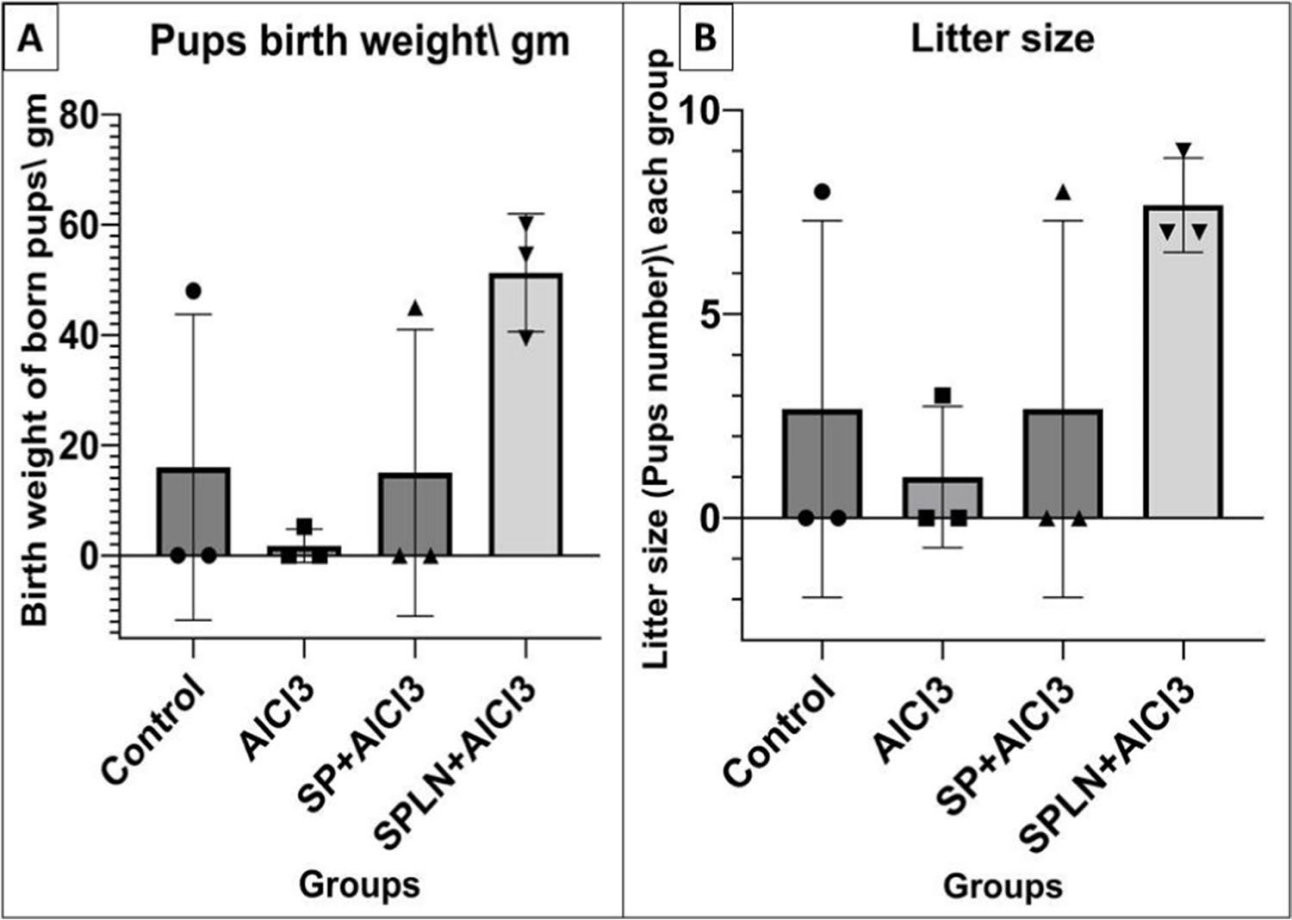
Effect of AlCl_3_, SP, and SPLN treatments on **A** pups’ birth weight and **B** litter size of births resulted from different treated groups. Results presented as mean and standard deviation of mean. Data graphed using GraphPad Prism10 were used for graphs by one-way analysis of variance (ANOVA) followed by post hoc test (Dunnett test)

Regarding fertility trait results (Figs. 3 and 4), it was noticed that females mated with AMGLN+AlCl_3_-treated males showed a marked reduction (*P < 0.05*) in their weights over weeks compared to control females, but AlCl_3_-treated mated females showed enhanced weights (*P < 0.05*) than the control females. On the other side, a notable decrease in the weights of control and AMG+AlCl_3_ (*P < 0.05*)- and AMGLN+AlCl_3_ (*P < 0.01*)-treated mated females were noticed when compared to AlCl_3_-treated mated females. Surprisingly, all mated females have no births. By noticing the control, AlCl_3_-, SP+AlCl_3_-, and SPLN+AlCl_3_-treated mated females; treatments had no effect on the weight of mated females. Furthermore, all females became pregnant, and an elevated litter size and birth weights in females mated with SPLN+ AlCl_3_-treated males recorded compared to other gps with absence of statistical difference.

Table 3 depicts the testicular levels of MDA and TAC in the various groups. Animals treated with AlCl_3_ were found to have a notably higher MDA and lower TAC than the control group (*P < 0.001*). The oral gavage of AMG, AMGLN, SP, and SPLN substantially reduced AlCl_3_-induced testicular oxidative stress *(P < 0.001*). Surprisingly, AMGLN and SPLN were excellent candidates for the highest results because they were successful in restoring MDA and TAC to controlled levels.

**Table 3:** Testicular levels of MDA and TAC in control and treated animals (mean ± SD)

Group	MDA (nmol/g testicular tissue)	TAC (mmol/g testicular tissue)
Control	0.92 ± 0.037	1.83 ± 0.070
AlCl_3_	2.50 ± 0.239 ^***^	0.64 ± 0.128 ^***^
AMG+AlCl_3_	1.88 ± 0.475 ^***#^	1.10 ± 0.360 ^***#^
AMGLN+AlCl_3_	1.12 ± 0.035^#^	1.69 ± 0.070 ^#^
SP+AlCl_3_	1.86 ± 0.048 ^***#^	1.12 ± 0.125 ^***#^
SPLN+AlCl_3_	1.21 ± 0.024 ^#^	1.59 ± 0.035 ^#^

*SD* standard deviation, *AlCl*_*3*_ aluminum chloride, *AMG* amygdalin, *AMGLN* amygdalin nanoparticles, *SP* spirulina, *SPLN* spirulina nanoparticles, *MDA* malondialdehyde, *TAC* total antioxidant capacity

^***^p < 0.001 versus normal control group

^#^*p* < 0.001 versus AlCl_3_ group

Using one-way ANOVA followed by Dunnett post hoc test

As shown in Table 4, AlCl_3_ induced marked decrement in the serum testosterone levels than the control group *(P < 0.001*). Interestingly, these negative effects of AlCl_3_ were greatly amended with AMG, AMGLN, SP, and SPLN treatments (*P < 0.001*). Furthermore, AMGLN and SPLN achieved the best findings. The quality of semen was greatly deteriorated with AlCl_3_. In this context, sperm motility, viability, and concentration were decreased, while the percentages of total sperm abnormalities were increased (*P < 0.001*) as opposed to the control group. These negative effects on semen quality were mitigated when AlCl_3_ were co-administered with AMG, AMGLN, SP, and SPLN (*P < 0.001*) in comparison to AlCl_3_ group. The most surprising discovery was that AMGLN and SPLN were effective treatments for reverting deviations in percentages of individual sperm motility, sperm concentration, sperm viability, and total sperm abnormalities to control levels.

**Table 4:** Serum levels of testosterone and semen quality in control and treated animals (mean ± SD)

Group	Testosterone ng/ml	Individual motility %	Sperm concentration × 10^6^/ml	Sperm viability %	Total sperm abnormalities %
Control	0.76 ± 0.14	82.33 ± 1.86	16.00 ± 1.79	90.83 ± 2.04	7.16 ± 1.17
AlCl_3_	0.21 ± 0.05 ^***^	53.00 ± 2.37 ^***^	6.67 ± 1.21 ^***^	62.50 ± 1.76^***^	33.17 ± 2.64 ^***^
AMG+AlCl_3_	1.36 ± 0.52 ^***#^	72.00 ± 8.46 ^***#^	11.57 ± 3.60^*#^	78.42 ± 7.82^***#^	15.14 ± 4.35^***#^
AMGLN+AlCl_3_	1.82 ± 0.05 ^***#^	85.83 ± 3.19 ^#^	14.00 ± 1.41^#^	90.00 ± 1.41 ^#^	8.17 ± 1.47 ^#^
SP+AlCl_3_	2.37 ± 0.13 ^***#^	73.50 ± 2.59 ^*#^	10.83 ± 1.47 ^***$^	84.67 ± 2.65^*#^	13.17 ± 1.47 ^***#^
SPLN+AlCl_3_	2.75 ± 0.07 ^***#^	85.33 ± 3.33 ^#^	14.50 ± 1.05 ^#^	91.67 ± 1.63 ^#^	7.83 ± 1.47 ^#^

*SD* standard deviation, *AlCl*_*3*_ aluminum chloride, *AMG* amygdalin, *AMGLN* amygdalin nanoparticles, *SP* spirulina, *SPLN* spirulina nanoparticles

^***^p < 0.001, ^*^p < 0.05 versus normal control group

^#^*p* < 0.001, ^$^*p* < 0.05 versus AlCl_3_ group

Using one-way ANOVA followed by Dunnett post hoc test

**Fig. 5:**
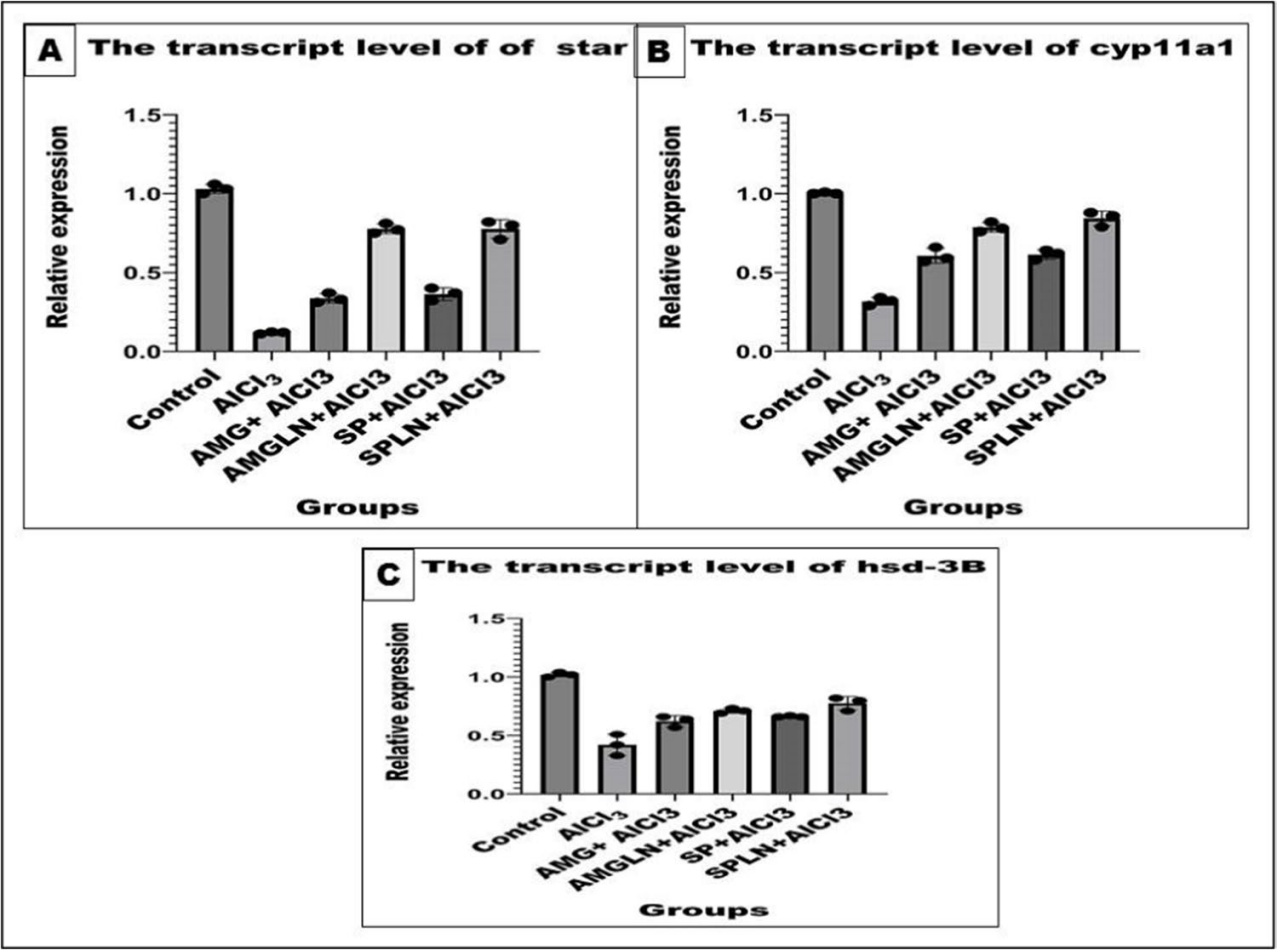
Testicular mRNA expression levels of CYP11A1, HSD-3b, and StAR in different groups. Results presented as mean and standard deviation of mean. Data graphed using GraphPad Prism10 were used for graphs by one-way analysis of variance (ANOVA) followed by post hoc test (Dunnett test)

As shown in Fig. 5, AlCl_3_ administration provoked marked down-regulation of the testicular mRNA expression levels of CYP11A1, HSD-3b, and StAR. It has been noticed that all treatments potentially counteracted the negative effects of AlCl_3_ and upregulated obviously the testicular mRNA expression levels of the target genes (*p < 0.05*). However, the most potent protection was exhibited by the nano-forms of both treatments compared to the macro-forms.

**Fig. 6:**
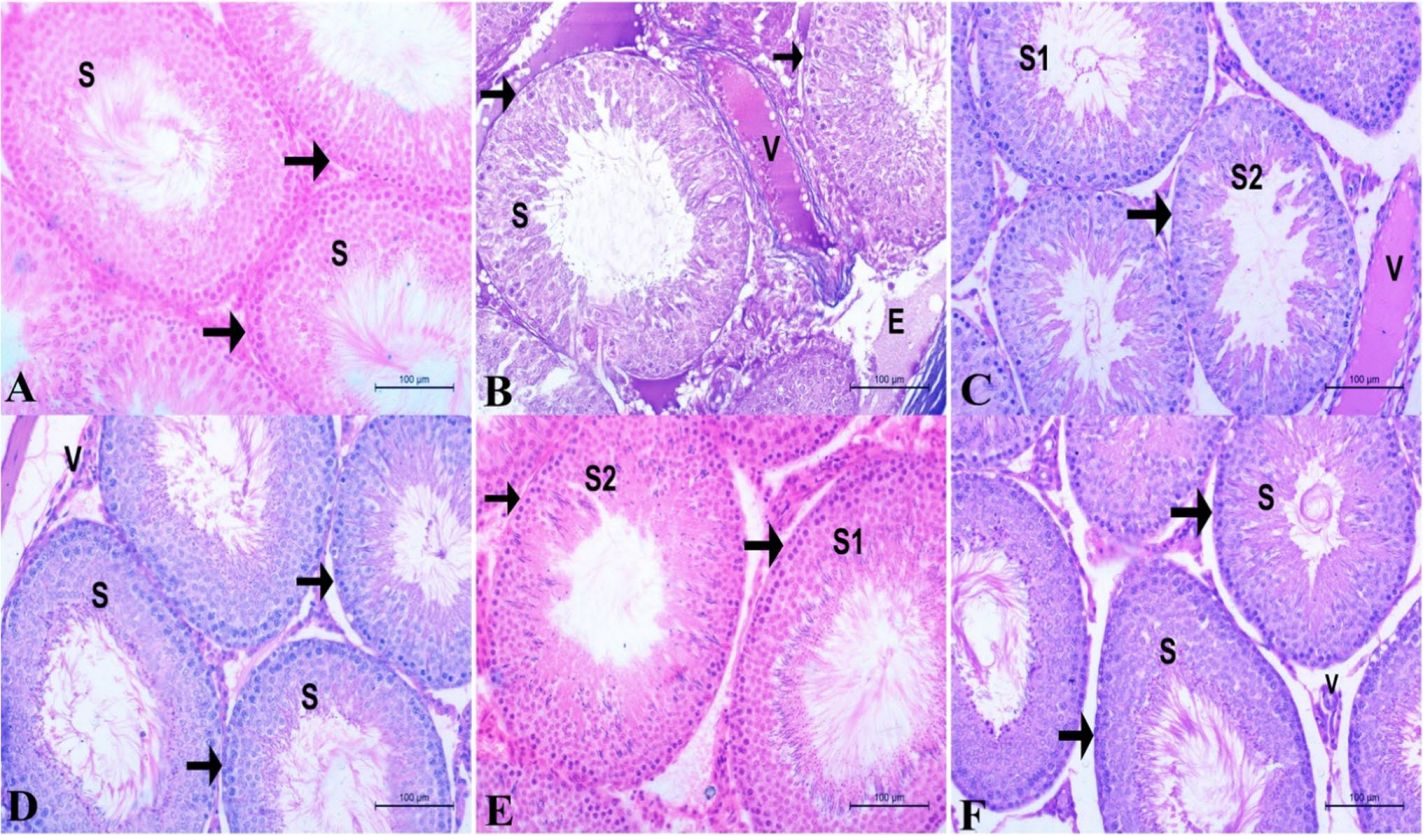
A photomicrograph of testes in adult male albino rats of the different tested groups showing the following: **A** Control group showing normal seminiferous tubules (S) enclosed by normal, thin basal membrane (arrow) and lined with normal, highly proliferative spermatogenic cells as well as huge amount of spermatid. The interstitial tissue between the tubules is containing normal blood vessels and active Leydig cells. **B** AlCl_3_-treated group showing seminiferous tubules (S) surrounded by thick basement membrane (arrow) and lined with spermatogenic cells suffered from degeneration and apoptosis as well as disappearance of spermatid and sperms. Note, the interstitial tissue contained congested blood vessels (V), marked degree of edema (E), and degenerated inactive Leydig cells. **C** AMG+AlCl_3_ group showing normal seminiferous tubules (S1) and other seminiferous tubules (S2) enclosed by thick basal membrane (arrow) and lined with spermatogenic cells with mild degenerative changes as well as few amounts of spermatid and sperms. Note, the interstitial tissue is containing highly congested blood vessels (V) and the active Leydig cells increased. **D** AMGLN+AlCl_3_ group showing normal seminiferous tubules (S) surrounded by normal basal membrane (arrow) and lined with normal spermatogenic cells as well as containing considerable amount of spermatid and sperms. The normal interstitial tissue is containing normal blood vessels (V) and active Leydig cells increased. **E** SP+AlCl_3_ group showing normal seminiferous tubules (S1) enclosed by thin basal lamina (arrow) containing normal spermatogenic cells and huge amount of spermatid and sperms, while other seminiferous tubules (S2) appeared less active containing few sperms. The interstitial tissue containing less congested blood capillaries, inactive Leydig cells and active Leydig cells increased. **F** SPLN+AlCl_3_ group showing normal seminiferous tubules (S) lined with highly proliferative spermatogenic cells rest on thin basal lamina (arrow) containing huge amount of spermatid and sperms in its lumen. The interstitial tissue is containing normal blood vessels and highly active Leydig cells. H&E stain ×200

In Fig. 6, the testicular tissue in the control group appeared normal with seminiferous tubules enclosed by normal, thin basal membrane and lined with normal, highly proliferative spermatogenic cells resulting huge amount of spermatid. The interstitial tissue between the tubules is containing normal blood vessels and active Leydig cells (Fig. 6A). By administration of aluminum chloride in AlCl_3_ group, there was a marked alteration of the testicular tissue architecture appearing in the form of thickening of the basement membrane which encloses the seminiferous tubules, the spermatogenic cells which lined the tubules appeared to be suffering from degeneration and apoptosis, and the disappearance of spermatid and sperms in its lumen. The interstitial tissue between these tubules contained congested blood vessels, marked degree of edema, and degenerated inactive Leydig cells (Fig. 6B). By using different treatments, there was a marked amelioration in the testicular tissue in different treated groups AMG, AMGLN, SP, and SPLN in order the ameliorative effect appeared as thinning of the basal membrane of the tubules, disappearance of the degenerative changes, increase the amount of spermatid and sperms as well as disappearance of vascular changes in the interstitial tissue in addition to increase the activity of the Leydig cells (Fig. 6C, D, E, and F).

## Discussion

Successfully, AMG-loaded niosome formulation was prepared. Due to a linear relationship between AMG concentration and absorbance (*R*^2^ = 0.998), the procedure described by Sohail et al. was found to be accurate for quantifying AMG. Literature review and pre-formulation investigations were used to select the best niosome formulation for in vitro and in vivo characterizations (Kazi et al., 2010; Chauhan & Bhatt, 2019; Chaw & Ah Kim, 2013; Waddad et al., 2013). Using HLB, the polarity strength of surfactants may be expressed quantitatively, allowing for the selection of appropriate surfactants to form niosomes with high physical stability (Nowroozi et al., 2018; Bnyan et al., 2018; Homaei, 2016). Span 60 and Tween 60 were chosen as non-ionic surfactants because of the lengthy alkyl chains that made it possible to create small niosomes with a high %EE and a rigid vesicular membrane (Manosroi et al., 2003; Bnyan et al., 2018; Waddad et al., 2013; Abdelbary & El-Gendy, 2008). A pre-formulation investigation revealed that an increase in HLB values was associated with a significant (*p* < 0.05) increase in %EE and particle size. High encapsulation of AMG was achieved using Tween 60–based formulations because of the hydrophilicity of AMG, the high HLB, and the high surface free energy of Tween 60 (Manosroi et al., 2003; Nowroozi et al., 2018; Bnyan et al., 2018; Waddad et al., 2013). Span 60’s high hydrophobicity led to a decrease in %EE and vesicle size (Waddad et al., 2013). Waddad et al. and Nowroozi et al. both found similar results (Nowroozi et al., 2018; Waddad et al., 2013). According to preliminary research, there is a positive correlation between cholesterol concentration and both %EE and particle size. Cholesterol’s ability to decrease surface free energy and increase bilayer hydrophobicity and stiffness contributed to the observed results of less leaky and more stable vesicles (Manosroi et al., 2003; Nowroozi et al., 2018; Waddad et al., 2013; Homaei, 2016). These results agreed with those found by Chaw et al. and Waddad et al. (Chaw & Ah Kim, 2013; Waddad et al., 2013). At the molar ratio studied, all AMGLN formulations with DDP showed a higher %EE and smaller particle size than those without DDP. DDP is a charge inducer utilized in the creation of niosomes, causing them to display a very negative zeta potential value and, hence, repulsive forces with the skin’s surface (Bnyan et al., 2018; Waddad et al., 2013). There was agreement between these results and those of Waddad et al. (Waddad et al., 2013). Niosomal formulations with a low PDI exhibited a homogeneous niosome with a low interfacial tension and no aggregation tendencies (Nowroozi et al., 2018; Bnyan et al., 2018; Waddad et al., 2013). The negative charge of the niosomal formulations produced electrostatic repulsions between vesicles, giving stable vesicles (Bnyan et al., 2018; Chaw & Ah Kim, 2013; Gamal et al., 2021; Shuwaili et al., 2016).

The male rats’ body weight obtained data run in consistent with the previous studies which reported a marked reduction in body weight of rats treated with AlCl_3_ (Yang et al., 2020; Buraimoh & Ojo, 2014; Justin-Thenmozhi et al., 2018). The reduced body weight as a result of AMG+AlCl_3_ and AMGLN+AlCl_3_ oral gavage may be attributed to AlCl_3_ toxicity besides absence of negative effect of them on body weight which run in agreement with (Dogru et al., 2017; Kovacova et al., 2020). However, the ability of SP+AlCl_3_ and SPLN+AlCl_3_ to ameliorate the weight loss induced by AlCl_3_ may be attributed to its content of highly digestible nutrients, vitamins, and minerals as previously reported by (Barkallah et al., 2020; Fouda & Ismail, 2017; Farag et al., 2016).

Regarding fertility trait results, it was noticed that females mated with AMG+AlCl_3_- and AMGLN+AlCl_3_-treated males showed a reduction in weight gain compared to control and AlCl_3_ gps, as well as no births were recorded. From these observations, due to the pregnancy percentage and after anatomical investigations macroscopically for the uterus, the feti were appeared to be absorbed inside the uterus. That might be because the prolonged administrations of amygdalin counteract and affect the fertility traits, besides the pregnancy rate not changing in the spermatozoa or male reproductive system. The impact of AMG on animal reproductive processes has been studied before (Tanyildizi & Bozkurt, 2004; Halenár et al., 2016). The outcomes of oral ingestion of AMG were assessed previously and induce reduced considerable reduction in the spermatozoa motility across time and dosage, as well as caused progressive motility. The hyaluronidase activity was strongly decreased by low doses of AMG (*P < 0.01*) as was also shown in a prior work (Tanyildizi & Bozkurt, 2004). In addition, most of the cyanogenic glycoside present in many plants markedly reduced the mobility of bull spermatozoa. Infertile males had considerably lower DNA polymerase alpha, beta, and gamma activities than healthy controls, according to a previous research (Fujisawa et al., 1988). Furthermore, amygdalin glycoside dose-dependently decreased the activity of rat DNA polymerase beta, according to Mizushina et al. (Mizushina et al., 1999). According to earlier research, all spermatozoa were immobile and dose-dependently lost their motility at 10 min (Tanyildizi & Bozkurt, 2004). The *in vitro* research by Halenár et al. (Halenár et al., 2016) indicates that short-term AMG supplementation does not have a deleterious impact on the survival of spermatozoa *in vitro*. It has been proposed that the first chemical released from AMG may be glucose, which may then promote the mitochondrial metabolism and then the motility activity of the spermatozoa (Halenár et al., 2016).

According to a recent research, AMG may have a dose-dependent effect on testicular tissue, exhibiting an intriguing paradox where low dosages may enhance the oxidative balance while large levels may jeopardize this sensitive environment (Albogami et al., 2020). So, based on this study and other research in our investigation, short-term ingestion of AMG at 300 mg/kg body weight in concentrated or nano-form did not confirm amygdalin’s toxicity on spermatozoa in vivo testing, but it did alter pregnancy or reproductive features.

On the other side, females mated with SP+AlCl_3_- and SPLN+AlCl_3_-treated male results run in parallel with Fouda et al. (Fouda & Ismail, 2017) who reported that SP did not differ in their effect on reproduction from control females and this was noticed in our results, although the administration of SPLN showed a superior effect on nanoparticles in improving the semen quality of male rats and consequently increasing pregnancy rate, birth weight, and litter size of the mated females.

One of the mechanisms that AlCl_3_ impairs testicular function is through induction of oxidative stress in the testes (Güvenç et al., 2020). Our data indicated that AlCl_3_ treatment provoked oxidative stress in the testes by increasing MDA and decreasing TAC. It has been reported that giving AlCl_3_ for 90 days resulted in elevation of MDA while suppressing catalase and superoxide dismutase (Yu et al., 2019). Similarly, AlCl_3_ increased MDA levels in the testes while decreasing antioxidant enzymes (Mohammad et al., 2015). Interestingly, AMG and AMGLN when co-administrated with AlCl_3_ notably alleviated the oxidative stress in testes. In this regard, AMG was found to mitigate the methotrexate-induced testicular oxidative challenges by eliminating the testicular thiobarbituric acid-reactive substances and enriching the testis with enzymatic antioxidants (Felemban et al., 2020). Amygdalin at dose of 100 mg/kg improved testicular oxidative status by elevating mRNA expression of antioxidants including glutathione peroxidase and superoxide dismutase while reducing lipid peroxidation (Albogami et al., 2020). As far as we are aware, there have been no research conducted on the AMGLN antioxidant ability. As a result, the superior antioxidant activity of AMGLN is attributed to the advantage of nanoparticles in performing tunable catalytic and redox capabilities, as well as their ability to bounce between multiple oxidation states (Lushchak et al., 2018).

Surprisingly, SP and SPLN crucially reduced the negative effects of AlCl_3_ and improved the oxidative markers in the testes. Furthermore, the improving action of SPLN surpasses the conventional form. In this regard, due to the inclusion of biologically active components that have powerful antioxidant effects such as polysaccharides and carotenoids, SP has been demonstrated to have antioxidant potential (Han et al., 2021). Rats subjected to a strength exercise regimen displayed substantial oxidative damage, as evidenced by raised MDA and diminished TAC, which was substantially lowered by SP (Brito et al., 2020). Furthermore, SPLN markedly alleviated the Ehrlich solid tumor (EST)–induced oxidative damage in hepatic tissue (Alheeti et al., 2021). Modified *Spirulina maxima* pectin nanoparticles enhanced porcine oocyte maturation in vitro by decreasing the reactive oxygen species and elevating the glutathione levels (Roy et al., 2021). The aforementioned findings corroborated our findings about SP or SPLN robust antioxidant effects. The antioxidant superiority of SPLN over SP may possibly be ascribed to the beneficial effects of nano-antioxidants when confronted with diverse oxidation states (Lushchak et al., 2018).

In the present study, serum testosterone levels had been markedly reduced with AlCl_3_ treatment. In this regard, a study of Pandey and Jain (Pandey & Jain, 2017) found that giving rat AlCl_3_ for 60 days decreased the serum level of testosterone. Additionally, administrating rats for 70 days also was observed to have negative effects on testosterone production (Yousef & Salama, 2009; Türk et al., 2021). The reduction in serum testosterone levels caused by AlCl_3_ could be due to atrophy of Leydig cells (Pandey & Jain, 2017). Furthermore, these negative effects of AlCl_3_ on testosterone production could be explained in light of our data regarding gene analysis that illustrated marked reduction of the testicular mRNA expression levels of CYP11A1, HSD-3b, and StAR, all of which are required for steroidogenesis (Manna et al., 2009; Mehanna et al., 2022). In this regard, AlCl_3_ decreased the number of Leydig cells while suppressing the mRNA expression of 3beta-hydroxysteroid dehydrogenase (3-HSD), 17-HSD, steroidogenic acute regulatory protein (STAR), and cholesterol side-chain cleavage enzyme (P450scc) (Mohammad et al., 2015). A study of Dong et al. (Dong et al., 2016) supported our results and reported that treating guinea pigs with Al and fluoride reduced testosterone production by suppressing the mRNA expressions of P450scc and StAR. Furthermore, the oxidative stress triggered with AlCl_3_ in the testes could be also a contributing factor to its negative influence on testosterone biosynthesis (Mohammad et al., 2015).

Spermatogenesis and consequently for sperm cells’ quality to be normal, testosterone is required. A lack of testosterone inhibits meiotic division and impedes progression of spermatogenesis beyond this stage (Walker et al., 2015). Our findings indicated that sperm cell quality was markedly reduced following AlCl_3_ treatment. These results are in tandem with studies of (Odo et al., 2021; Olusola, 2019) who reported negative effect of AlCl_3_ on male reproductive physiology via decreasing the serum testosterone levels, impairment of sperm motility, decreasing viability, increasing sperm deformities. In this concern, AlCl_3_ treatment resulted in a vital decrease in sperm viability, concentration, and motility, as well as an increase in the percentage of defective sperms (Felemban et al., 2020). The authors of that study explained AlCl_3_’s deleterious effects on sperm quality by inducing oxidative stress in the testes, which is in line with our findings Table 5.

**Table 5:** Histopathological lesions scoring of testis in the experimental groups

Group	Testis				
	Degeneration of spermatogenic cells	Depletion of sperms	Thickening of the basal membrane	Congestion of testicular vessels	Edema of the interstitial tissue
Control	**0**	**0**	**0**	**0**	**0**
AlCl_3_	**2**	**3**	**3**	**4**	**3**
AMG+AlCl_3_	**1**	**1**	**1**	**2**	**1**
AMGLN+AlCl_3_	**0**	**0**	**0**	**0**	**0**
SP+AlCl_3_	**0**	**1**	**0**	**1**	**0**
SPLN+AlCl_3_	**0**	**0**	**0**	**0**	**0**

Histopathological scoring of testicular tissue injury was scored in degrees as follows: 0 = no change; 1 ≤ 25% tissue damage; 2 = 26–50% tissue damage; 3 = 51–75% tissue damage; 4 = 76–100% tissue damage. Bold entries are streamly significant at *P*<0.05

On the other side, serum testosterone levels and sperm quality were markedly improved with the AMG_,_ AMGLN, SP, and SPLN. Furthermore, AMGLN and SPLN were successful in restoring sperm quality to a level that was close to that of the control group. The obtained data were in accordance with those of Felemban et al. (Felemban et al., 2020) who reported that AMG can counteract the effects of methotrexate on testosterone synthesis. The authors attributed AMG beneficial effects to its robust antioxidant properties, which coincide with our findings. Concerning the improving effect of AMG on sperm quality, Fan et al. (Fan et al., 2016) reported that AMG treatment eased the harmful effects of acetate on sperm quality by improving sperm density, reducing sperm abnormalities, and boosting the spermatogenesis process via increasing the energy metabolism enzymatic activity. Fan and his colleagues explained such an improvement in spermatogenesis by AMG ability to increase serum testosterone levels, enrich the testicular environment with enzymatic antioxidants (superoxide dismutase), and eliminate pro-oxidant markers, which confirm our results. Interestingly, SP was observed to ameliorate the deleterious effects of lead acetate–induced dysfunction of testes by amending the serum testosterone level and the oxidative stress to control level (Ibrahim et al., 2021) . Furthermore, it has been found that SP is an effective treatment for protecting mouse testicular function against bifenthrin reprotoxicity while maintaining normal testosterone levels and sperm quality (Barkallah et al., 2020). As far as we are aware, there have been no research done to investigate the effect of AMGLN and SPLN on testosterone production and spermatogenesis; however, the beneficial effects may be attributed to their high antioxidant activities (Felemban et al., 2020; Elabd et al., 2020).

In addition, upon our gene analysis data, we speculated that the favorable effects of AMG_,_ AMGLN, SP, and SPLN against AlCl_3_-induced reduction in testosterone levels and, consequently, sperm quality could be accounted for their potential to restore the mRNA expression levels of CYP11A1, HSD-3b, and StAR. The study of Khalil et al. (Khalil et al., 2021) found that testosterone and sperm quality that were reduced in diabetic rats were restored to normal levels when specific steroidogenic genes including 3βHSD, 17βHSD, StAR, and CYP11A1 recovered to controlled levels by myristic acid. The key gene implicated in the testosterone synthetic pathway (3β-HSD) was found to be restored to controlled level when SP was co-administered with bifenthrin, keeping testosterone and sperm quality at normal state (Barkallah et al., 2020). The transcriptional regulation of STAR, CYP11A1, and 3 HSD-3b are regulatory points in the steroidogenesis which is greatly affected by the lack of storage of the steroid hormones (Noshy et al., 2022; King & LaVoie, 2012)

In this investigation, we examined some genes connected to steroidogenesis in the testes. The rate-limiting phase in steroid biosynthesis is regulated by the StAR (Steroidogenic Acute Regulatory) gene. It controls the transportation of cholesterol from the outer to the inner mitochondrial membrane, which is crucial for controlling the synthesis of steroid hormones (Manna et al., 2009). A protein-coding gene called CYP11A1 (Cytochrome P450 Family 11 Subfamily A Member 1) catalyzes the transformation of cholesterol into pregnenolone, the precursor to the majority of steroid hormones (Chen et al., 2021). The protein-coding gene HSD-3b catalyzes the oxidative conversion of delta-5-3-beta-hydroxysteroid precursors into delta-4-ketosteroids, which results in the generation of all classes of steroid hormones. The enzyme cytochrome P450 (CYP11A1) in the mitochondria catalyzes the conversion of cholesterol side chains to pregnenolone, which starts the process of steroidogenesis. The mitochondrial transport of cholesterol is facilitated by StAR (Mehanna et al., 2022). A number of oxidative enzymes found in both the mitochondria and endoplasmic reticulum then catalyze the conversion of pregnenolone into other steroids. The resulting functional steroids in a given gland or tissue are determined by the accessibility of these enzymes in a given tissue (Noshy et al., 2022). The impact of CYP11A1, HSD-3b, and StAR transcript levels was down-regulated in testicular tissues following the AlCl_3_ administration. However, experimental mice exposed to AlCl3 have altered testicular development and testosterone synthesis (Lokman et al., 2022). The injurious effects provoked by the AlCl3 may be attributed to the increased mitochondrial oxidative stress and disorders of the mitochondrial energy metabolism. Adenosine triphosphate (ATP) production, Leydig cell steroidogenesis, and other components of male reproductive processes are all regulated by testicular mitochondria (Ibrahim et al., 2019).

It was obvious that administration of AlCl_3_ markedly affected the rats’ testicular tissue via presence of degenerative changes in the seminiferous tubules and interstitial tissue (Pandey & Jain, 2017; Boudou et al., 2020; Buraimoh et al., 2012). The protective roles of AMG and SP were evident via restoring the testicular tissue to the normal appearance and improving the histological architecture of testicular tissue (Barkallah et al., 2020; Albogami et al., 2020; Felemban et al., 2020; El-Desoky et al., 2013).

## Conclusion

The goals of the present investigation were to assess the reprotoxicity of AlCl_3_ in adult male rats utilizing several testicular tissue evaluations, including histopathology, oxidative enzyme activity monitoring, antioxidant serum enzyme estimation, and changes in numerous testicular gene expression. In this pathology-related animal model, more research should be done into the possible protective benefits of SP and amygdalin in conventional and niosomal forms against the reprotoxicity generated by AlCl_3_. In addition to successful loading on the nanoparticles of niosomes, we provide a wide range of reliable data to back up the claim that SP or amygdalin may be used as a palliative therapy to avoid infertility in populations exposed to high concentrations of pesticides containing aluminum chloride.

### Supplementary information

ESM 1 (XLSX 1704 kb)

## Declarations

### Data availability

Will be available after acceptance and publishing in this journal.
